# Roles of extracellular adenosine triphosphate on the functions of periodontal ligament cells

**DOI:** 10.1038/s41405-023-00147-7

**Published:** 2023-07-08

**Authors:** Maythwe Kyawsoewin, Jeeranan Manokawinchoke, Worachat Namangkalakul, Hiroshi Egusa, Phoonsuk Limraksasin, Thanaphum Osathanon

**Affiliations:** 1 Dental Stem Cell Biology Research Unit and Department of Anatomy, Faculty of Dentistry, Chulalongkorn University, Bangkok, Thailand; 2 Center of Excellence for Regenerative Dentistry, Chulalongkorn University, Bangkok, Thailand; 3 Division of Molecular and Regenerative Prosthodontics, Tohoku University Graduate School of Dentistry, Sendai, Miyagi Japan; 4 Center for Advanced Stem Cell and Regenerative Research, Tohoku University Graduate School of Dentistry, Sendai, Miyagi Japan

**Keywords:** Periodontitis, Gingivitis

## Abstract

**Objective:**

Adenosine triphosphate (ATP) is an essential nucleotide that is normally present in both intracellular and extracellular compartments. Extracellular ATP (eATP) has a pivotal role in both physiological and pathological processes of periodontal ligament tissues. Here, this review aimed to explore the various functions of eATP that are involved in the control of behaviours and functions of periodontal ligament cells.

**Methods:**

To identify the included publications for review, the articles were searched in PubMed (MEDLINE) and SCOPUS with the keywords of adenosine triphosphate and periodontal ligament cells. Thirteen publications were used as the main publications for discussion in the present review.

**Results:**

eATP has been implicated as a potent stimulator for inflammation initiation in periodontal tissues. It also plays a role in proliferation, differentiation, remodelling, and immunosuppressive functions of periodontal ligament cells. Yet, eATP has diverse functions in regulating periodontal tissue homeostasis and regeneration.

**Conclusion:**

eATP may provide a new prospect for periodontal tissue healing as well as treatment of periodontal disease especially periodontitis. It may be utilized as a useful therapeutic tool for future periodontal regeneration therapy.

## Introduction

Periodontal ligament cells (PDLCs) possess stem cells that have similar mesenchymal stem cell characteristic features. PDLCs can be differentiated into different cell types like cementoblasts, fibroblasts, and osteoblasts [1, 2]. As PDLCs have the ability to balance between new cell formation by proliferation and cell death, PDLCs have a cell renewal capacity [3]. Therefore, PDLCs may be the main cell source and a promising target approach for periodontal regeneration therapy. But its utilisation alone has some limitations; some conditions, like inflammatory environments, change the characteristic features of resident periodontal ligament cells [2, 4, 5]. Growth factors and molecular activities are required to stimulate resident PDLCs for effective periodontal regeneration. Many factors like VEGF, FGF2, IL1β, and IL12 participate in different periodontal regeneration stages to synergise periodontal regeneration and the regenerative ability [6–8]. Despite many factors and molecules involved in the periodontal regeneration process, we targeted adenosine triphosphate and explored its effects on PDLCs functions for this review.

Adenosine triphosphate (ATP) is an essential nucleotide and is normally found intracellularly and extracellularly. Both forms of ATP are involved in the physiological as well as pathological processes of various cell types. The amount of ATP in the extracellular environment during physiological conditions is relatively low [9]. Some conditions like mechanical stress induced the release of ATP into the extracellular environment by PDLCs [10–12]. The released ATP has different functions, such as proliferation, differentiation, and inflammatory response on different cell types. eATP induces proliferation through PKC, PI_3_K/Akt, and MAPK signalling pathways in mouse embryonic stem cells [13]. It also acts as a danger signal by inducing the release of pro-inflammatory cytokines like IL1β in MG-5 microglial cells and IL6 in human thyrocytes [14, 15]. It has immunosuppressive action by stimulating IDO expression in the bone marrow mesenchymal stem cells (BMSCs) [16]. Different functions of PDLCs have been implicated in the periodontal regeneration process. However, the effects of eATP on the functions as well as behavior of PDLCs have not been extensively reviewed. This review aims to evaluate the various impacts of eATP on the functions and properties of PDLCs.

## Methods

The articles were searched in PubMed (MEDLINE) and SCOPUS databases using keywords without published period limitation for this review. The keywords used for the search are [adenosine triphosphate AND periodontal ligament cells]. The authors examined and evaluated the title and abstracts of the articles for inclusion and exclusion criteria. The inclusion criteria were as follows; (1) full-text articles published in English or other language articles with available English abstracts, (2) articles demonstrating the effect of extracellular adenosine triphosphate related to functions of PDLCs, including inflammation, differentiation, immunomodulatory functions, and other functions of PDLCs. The exclusion criteria were (1) any study published in other languages, (2) articles related to the effect of adenosine triphosphate on other cell types rather than PDLCs (3) the study evaluating the effect of intracellular adenosine triphosphate.

## Results

**Fig. 1:**
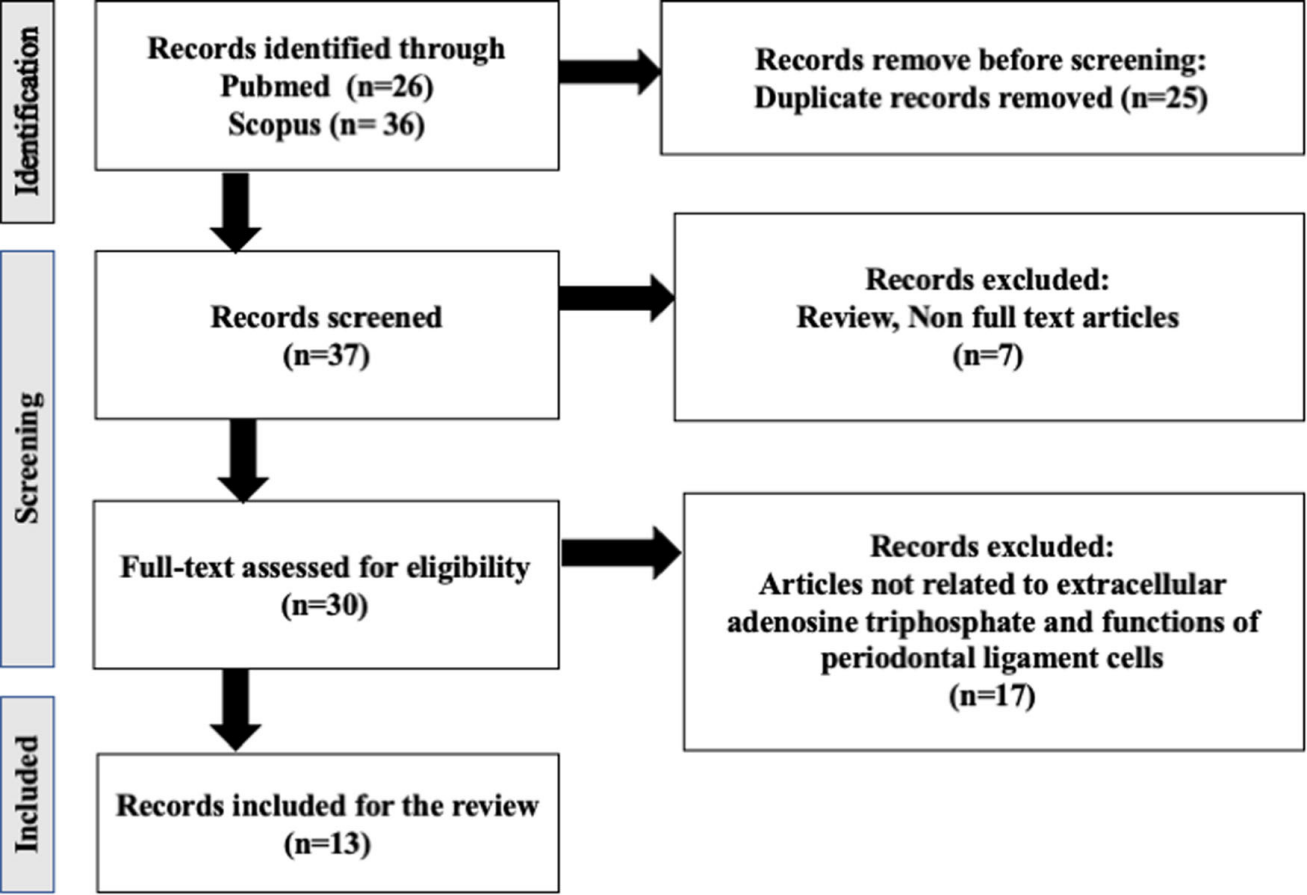
Flow chart illustrating the article selection. Articles are selected for this review by using keywords; adenosine triphosphate (ATP) and periodontal ligament cells (PDLCs).

37 original articles were found in search of the databases by using the described search procedure. According to inclusion and exclusion criteria, 7 non-English studies were removed, and we removed another 17 studies that are not related to extracellular adenosine triphosphate and functions of PDLCs. Final 13 studies were used for this review. We used Preferred Reporting Items for Systematic Reviews and Meta Analyses (PRISMA) for the selection of literature for this review. The flow of information through the different steps involved in the selection of studies for this review (PRISMA) is shown in Fig.1.

### Periodontal ligament cells (PDLCs)

The periodontal ligament is one of the tooth’s supporting tissues and is involved in the periodontium together with the cementum and alveolar bone. It is a fibrous tissue rich in vascular supply and connects the tooth cementum on one side and the alveolar bone on either side. As it is a tooth-supporting tissue, it plays a crucial role in maintaining tooth stability and tuning biological functions. One of the periodontal ligament’s major functions is maintaining periodontium homeostasis; for instance, it controls physiologic mechanical force during the masticatory function by transferring force to other supporting tissues of the tooth. The periodontal ligament can also play a key role in periodontal regeneration because it constitutes multiple cell types, such as cementum-forming cells (cementoblasts), bone-forming cells (osteoblasts), nerve cells, fibroblasts, and vascular endothelial cells. Thereby, the periodontal ligament becomes a major cell source for maintaining periodontal tissue homeostasis and regeneration [1, 17]. Periodontal ligament cells (PDLCs) are present in periodontal ligament tissues, which are in periodontal ligament space. PDLCs own stem cells (progenitor cells) and have similar mesenchymal stem cell characteristics and features. They have multiple lineage differentiation abilities that can be transformed into cementoblasts, fibroblasts, osteoblasts, and adipose cells [1, 2, 18, 19]. Beside mesodermal lineages, stem cells isolated from periodontal ligaments can also differentiate into ectodermal and endodermal lineages [20, 21]. PDL fibroblasts are capable of renewal of cells that can be adjusted between new cell formation by proliferation and number of cell loss through cell death and cell migration [3].

### Periodontal regeneration

The periodontal tissue regeneration process includes three consecutive phases: inflammation, proliferation, and remodelling. It is similar to other tissue healing processes [22]. However, some pathological and immunocompromised conditions like periodontitis imbalance the normal regeneration process that causes excessive and prolonged inflammatory phase, lack or delayed cell proliferation, and repairing phase leading to periodontal tissue destruction [23]. The final goal of periodontal regeneration is to form new functional periodontal tissue in place of the damaged periodontal tissue [24]. PDLCs may be the main cell source for periodontal regeneration by reason of their similar mesenchymal stem cell features like multilineage differentiation, proliferation, and self-renewal ability. In particular, PDLCs are easily accessible and expanded ex vivo [25]. Nevertheless, PDLCs could be used as a prioritized approach for regenerative treatment in various periodontal diseases. The success of regenerative treatment using PDLCs alone is very restricted because of some restrained conditions such as inflammatory conditions of host PDLCs [2, 4, 5]. Therefore, periodontal therapy needs other factors besides PDLC therapy for effective periodontal regeneration. Additional molecular activities are needed for the periodontal regeneration process by regulating in stages of the regeneration process; some molecules participate in cell proliferation, some in differentiation, some in control of the immune response, and some regulate the release of inflammatory mediators by PDLCs.

As a result of previous studies (as shown in Table 1), many factors participated in the stages of regeneration, in particular inflammation, immunoregulation, proliferation, differentiation, and maturation. Consequently, PDLCs possess stemness, proliferative, immunomodulatory, and differentiation properties; other factors boost the regenerative ability of PDLCs. Among many factors involved in the periodontal regeneration process, we emphasized the role of adenosine triphosphate in the functions of PDLCs in this review.

**Table 1:** Factors participate in the regulation of periodontal regeneration

No:	Factors	Functions	References
1.	VEGF	Enhances osteogenic differentiation by promoting alkaline phosphatase activity, mineralized structure formation and Runx2 expression in PDLSCs	[6]
2.	FGF2	Promotes progenitor cells proliferation	[6]
3.	rhBMP2	Regeneration of a mineralized layer mimicking dental cementum	[68]
4.	PDGF and TGFβ1	Enhances Periodontal healing by inducing PDLCs proliferation	[69]
5.	IL1β	Change LPS responsiveness PDLCs phenotype from osteoblastic characteristic phenotype	[7]
6.	IL6	Osteolytic factors and induces osteogenic differentiation	[70]
7.	IL12	Immunomodulatory function of PDLCs by inducing IFNγ, IDO, and HLA-G expression	[8]
8.	Poly I:C (TLR3 agonist)	Enhance the immunomodulatory properties of PDLCs by enhancing immunomodulatory molecules IFNγ, IDO	[71, 72]

### Adenosine triphosphate (ATP)

ATP is an essential nucleotide that is built up of a purine base (adenine), pentose sugar (ribose) and 3 phosphate groups. Adenine is attached to the carbon atom at 1’place, and 3 phosphate groups are attached at the 5’ place of ribose. The 3 phosphate groups which are attached to the carbon atom at 1’ place, are linked to ATP by high energy bonds. ATP is normally found in both intracellular and extracellular compartments. Intracellular ATP (iATP) functions as the intracellular energy source of the cells. eATP acts as an essential extracellular messenger [9]. Both iATP and eATP take part in physiologic and pathological conditions, involving different functions like inflammatory process, healing process, and immune responses of various cell types.

### Functions of intracellular ATP (iATP)

iATP usage promotes skin wound healing resulting in granulation tissue formation, re-epithelialization, and increased VEGF release [26]. iATP promotes wound healing of rabbits’ skin. It attracts the macrophage and inflammatory cells to the wound site and actuates the release of inflammatory cytokines: IL1β and TNFα, along with increases in VEGF expression, without the formation of hypertrophic scar in skin wounds of rabbits. Still, this study has limitations, and the detailed mechanism of the healing process may be specific to the species [27]. Mg-ATP encapsulated lipid vesicles treated at to wound site cause rapid granulation tissue regeneration, and new growth starts in less than 1 day [28]. These previous studies indicate the role of iATP in the wound healing process.

### Functions of extracellular ATP (eATP) and its receptors

The amount of eATP is relatively low (400–100 nM) under physiologic conditions [9]. Many situations like mechanical stress, hypoxia, and inflammation cause the release of ATP into the extracellular compartment by various cell types such as PDLCs, cardiomyocytes, and endothelial cells [11, 12, 29, 30]. eATP has different functions, but it depends on various factors like cell types and types of activated receptors [31]. It acts as a danger signal called Danger-Associated-Molecular-Pattern Molecule (DAMP). It binds and activates the purinergic receptors on the cell surfaces, then initiates the inflammatory signal cascade and regulates the immune response [32, 33]. As the released ATP cannot be entered into the cell membrane easily to precede intracellular signalling events, it would rather interact with purinergic P_2_ receptors on the cell surface.

Purinergic P_2_ receptors are usually related to ATP. It has 2 subtypes according to signaling properties: metabotropic P_2_Y receptors (P_2_YRs) and inotropic P_2_X receptors (P_2_XRs). P_2_Y receptors (P_2_YRs) are classical G-protein-coupled receptors expressed in mammalian cells are eight subtypes (P_2_Y_1_, P_2_Y_2_, P_2_Y_4_, P_2_Y_6_, P_2_Y_11_, P_2_Y_12_, P_2_Y_13_, P_2_Y_14_). P_2_Y receptors are activated by ATP, ADP, UTP, UDP, UDP glucose, and NAD (nicotinamide adenine dinucleotide). The intracellular signaling event of P_2_Y_1_, P_2_Y_2_, P_2_Y_4_, P_2_Y_6,_ and P_2_Y_11_ is related to PLC-IP_3_R signaling pathway resulting in increased intracellular Ca^2+^ level. P_2_Y_12_, P_2_Y_13_, P_2_Y_14_ receptors are mediated by AC-cAMP signaling pathway; ATP binds to these receptors leading to the inhibition of adenylyl cyclase (AC) and decreased intracellular cAMP levels [34, 35]. P_2_X receptors (P_2_XRs) are nucleotide-gated ion channel receptors with seven subtypes (P_2_X_1_, P_2_X_2_, P_2_X_3_, P_2_X_4_, P_2_X_5_, P_2_X_6_, P_2_X_7_) when ligand (nucleotide) gated ion channels P_2_X receptors are activated by ATP. These are homo/hetero-trimers, eATP binds to receptors, ion channels opened, K^+^ efflux and an influx of Ca^2+^ and Na^+^ occur resulting in increased intracellular calcium and membrane depolarization [36, 37].

eATP gives different functions to different cell types; one of the dependent factors is the types of purinergic receptors. Different receptor activation by ATP influences many cell functions including proliferation, inflammation, immune response, and others. eATP is involved in the suppression of endometrial stem cell proliferation and migration [38]. eATP-P_2_Y_1_ receptor activation reduces the proliferation of BMSCs [39]. On the other hand, eATP could induce mouse embryonic stem cell proliferation through PKC, PI_3_K/Akt, and MAPK signaling pathways. Therefore, eATP has different effects on cell proliferation in different environments through various signaling [13].

eATP plays a significant role in the control of inflammation of different cell types through diverse P_2_ receptor interactions. In MG-5 microglial cell lines, eATP induces the maturation and release of IL1β by enhancing IL1β converting enzyme/ caspase [14]. eATP also stimulates other pro-inflammatory cytokine IL6 release in human thyrocytes that plays role in the control of thyroid function. eATP dose-dependently induced IL6 release through P_2_Y receptor in human thyrocytes [15]. ATP can also induce anti-inflammatory cytokine IL10 expression and this induction is related to the crosstalk between P_2_Y_1_ and P_2_Y_11_ receptor activation in microglial cells. However, this induction effect is also dependent on intracellular Ca^2+^ release or the cAMP-activated PKA pathway [40]. In human blood cells, eATP-P_2_Y_11_ interaction maintains the balance of inflammatory mechanism by increasing IL10 and decreasing TNFα [41]. P_2_X_7_ receptor activation induces TNFα release by LPS-induced microglial cells of rat [42]. This receptor activation also induces inflammation by different mechanisms in different cell types; upregulates IL1β expression by stimulating NALP3 inflammasome and caspase 1 in *P.gingivalis* induced gingival epithelial cells [43], promotes IL6 expression by LPS pretreated primary human skin fibroblasts [44]. Another P_2_X receptor (P_2_X_5_) activation induces inflammasomes and IL1β secretion in murine osteoclasts [45].

Activation of P_2_Y_2_ and P_2_Y_4_ receptors by ATP regulates tumor growth and progression by inducing transcription factors, ERK1/2, p38 and JNK1 phosphorylation in MCF-7 cells [46]. eATP is involved in the regulation of human gingival tissue destruction by inhibiting IL1-induced matrix metalloproteinases (MMPs) expression via CD39 expression [47]. ATP-dependent P_2_X_3_ receptor activation increases endometrial pain by inducing neurogenic inflammation [48].

Various P_2_ receptors activation by ATP imparts in an immune response. eATP plays a regulatory role in the immune response by adjusting specific CD^4+^ T cells response by activation of different P_2_ receptors. 250 nM ATP upregulates the survival and proliferation of T lymphocytes by increasing the secretion of IL2. A higher dose 1 mM of eATP enhances apoptosis and inhibits activated CD^4+^ T cells functions through P_2_X_7_ receptor activation and enhances the proliferation of regulatory T cells by activation of P_2_Y_2_ receptors. Hence, the effect of eATP on specific CD^4+^ T cells response depends on the concentration of nucleotide [49]. eATP supports the immunoregulatory mechanism of dendritic cells. It inhibits Th_1_ cytokine IL12 and stimulates Th_2_ cytokines IL10. It also provides immunosuppressive action by inducing IDO expression through P_2_Y_11_ receptor in monocyte-derived dendritic cells primed with IFNγ [50]. ATP-P_2_ receptor activation has the full ability of immunosuppression by activating naïve T reg cells [51]. P_2_X_7_ receptor activation by 1000 μM eATP induces immunomodulatory cytokine IFNγ release in Japanese flounder head kidney cells [52]. eATP is involved in the immunosuppressive function of BMSCs primed with IFNγ by downregulating IDO expression via P_2_X_7_ receptor activation [16]. In conclusion, eATP has diverse functions of different cell subsets through various P_2_ receptors. Different functions of ATP that depend on various cell types and different receptor activation are described in Table 2.

**Table 2:** Different functions of ATP according to types of cells and receptor activation

No:	Receptors	Mechanism	Functions of ATP	Cell types	References
1.	P_2_	Immune response	Full activation of T reg cells	Naïve T reg cells	[51]
2.	P_2_Y	Inflammation	Induces IL6 release	human thyrocytes	[15]
3.	P_2_Y_1_	Proliferation	Decreases proliferation in early passage of culture (P0-P5)	BMSCs	[39]
4.	P_2_Y_1_ P_2_Y_11_	Inflammation	Elevation of IL10	Macroglia cells	[40]
5.	P_2_Y_11_	Inflammation	decrease TNFα release and upregulate IL10 production	Human blood cells	[41]
6.	P_2_Y_11_	Immune response	Promotes T cells immunosuppression by upregulating IDO expression	IFNγ primed monocyte derived dendritic cells	[50]
7.	P_2_Y_2_ P_2_Y_4_	Tumor growth and progression	Induces ERK1/2, p38 and JNK1 phosphorylation	MCF-7 breast cancer cells	[46]
8.	P_2_Y_2_	Immune response	Enhances proliferation of T reg cells	T cells	[49]
9.	P_2_X_3_	Pain	Induces neurogenic inflammation	Human endometriotic cells	[48]
10.	P_2_X_5_	Inflammation	activates inflammasomes and IL1β secretion	Murine osteoclasts	[45]
11.	P_2_X_7_	Inflammation	Promotes the production of TNFα	LPS induced rat macroglia cells	[42]
12.	P_2_X_7_	Inflammation	Increases IL1β expression	*P.gingivalis* induced gingival epithelial cells	[43]
13.	P_2_X_7_	Inflammation	Upregulates IL6	LPS pretreated primary human skin fibroblasts	[44]
14.	P_2_X_7_	Immune response	1 mM ATP induces apoptosis and inhibits activated CD^4+^ T cell function	T cells	[49]
15.	P_2_X_7_	Immune response	Induces the release of IFNγ	Japanese flounder head kidney cells	[52]
16.	P_2_X_7_	Immune response	induces immunosuppression by increasing IDO expression	BMSCs	[16]
17.	Not specified	Proliferation and migration	Suppress proliferation and migration	Endometrial stem cells	[38]
18.	Not specified	Proliferation	Induces proliferation	Mouse embryonic stem cell	[13]
19.	Not specified	Inflammation	Induces the maturation and release of IL1β	MG-5 microglial cell lines	[14]
20.	Not specified	Immune response	250 nM ATP supports survival and proliferation of T lymphocytes	T cells	[49]

As stated in Table 2, eATP has diverse effects on various functions according to different receptor activation and cell types. PDLCs have different types of purinergic receptors on the cell surface. Many previous studies proved that eATP and P_2_ receptor signalling had different functions on PDLCs.

### Roles of eATP in various functions of PDLCs

eATP has numerous effects on the functions of PDLCs. It depends on different receptor interactions. One or more subtypes of purinergic P_2_ receptors are found on all types of cells. Previous study’s result clarified P_2_X_7_, P_2_Y_1_, P_2_Y_2_, P_2_Y_4_, P_2_Y_6_ and P_2_Y_12_ receptors expression has been detected in the periodontal ligament cells, that was detected in the 9 days cultured conditions [53]. As ATP can bind to various P_2_ receptors, the interaction of eATP and P_2_ receptor signalling had different functions on PDLCs, including proliferative function, inflammatory response, immunosuppressive function, osteogenesis, bone destructive function, and other various functions. One receptor can involve in different mechanism of PDLCs’ functions, sometimes more than one receptor is involved in each function. Different roles of eATP affect the numerous functions of PDLCs through different signalling, as shown in Table 3.

**Table 3:** Roles of eATP in various functions of PDLCs.

No	Receptors	Mechanism	Functions of ATP	Conditions	References
1.		Proliferative action	Suppress PDLCs proliferation	PDLCs	[54]
2.	P_2_X_7_	Inflammatory chemokines	Induces IL8 and CCL 20 release	PDLCs	[55]
3.	P_2_X_7_	Pro-inflammatory cytokines	Induce release of IL1β	Mechanical stress induced PDLCs	[12, 56]
4.	P_2_X_7_	Immunosuppressive cytokines	Induces IDO and IFN *γ* expression	PDLCs	[57]
5.	P_2_X_7_	Osteogenic markers	Reduces osteogenic differentiation	Inflammatory mediated PDLCs	[58]
6.	P_2_X_7_	Osteogenic markers	Enhances Runx2 and OCN expression	PDLCs	[59]
7.	P_2_Y_1_	Osteogenic factor	Stimulates BMP-9 synthesis	Cyclic tensile stress induced PDLCs	[60]
8.	P_2_Y_1_	Osteoclast differentiation marker	Promotes RANKL expression through the P_2_Y_1_ cyclooxygenase pathway	Mechanical stress induced PDLCs	[61]
9.	P_2_Y_1_	Bone resorption factor	Induces osteopontin expression	Mechanical stress induced PDLCs	[62]
10.	P_2_Y_4_ P_2_Y_6_	Remodeling factor	Induces ERK phosphorylation	Stress induced PDLCs	[63]
11.		Behavior of PDLCs	Regulates function and behavior of PDLCs through ATP-connexin 43 channels	Continuous compressive stress induced PDLCs	[11]
12.		Pain factor	Control nociceptive pain due to orthodontic tooth movement	Mechanical stimulated PDLCs	[64]

### Effects of eATP on the proliferation of PDLCs

eATP modulates the proliferation of different cell types through specific purinergic receptors. Extracellular ATP and slowly hydrolyzable ATP (ATP*γ*S) suppress the PDLCs proliferation but not the same mechanism. ATP induced PDLCs growth arrest by increasing p21^WAF1/cip1^ that regulates cell proliferation by inhibiting the cell cycle through the cyclin kinase pathway. Extracellular ATP*γ*S induced cellular apoptotic responses. Ectonucleotidases including CD39 which are present in serum rescued the suppressive effect of PDLCs proliferation by ATP and ATP*γ*S [54].

### Effects of eATP on the inflammatory function of PDLCs

Mechanical stress induced the release of ATP by PDLCs. The released ATP activates specific purinergic P_2_ receptors on the cell surface and has been shown to regulate the trigger of pro-inflammatory cytokines/ chemokines. ATP induces the maturation or the release of pro-inflammatory cytokines /chemokines by PDLSCs. P_2_X_7_ receptor agonist (BzATP) enhanced the release of IL8 and CCL20 without influencing cell viability. Specific P_2_X_7_ receptor irreversible inhibitor, oxidized ATP (oATP) or A-74003 counteracted the eATP-induced IL8 and CCL 20 release. This inductive effect is followed by an increase in intracellular Ca^2+^ signalling. Generally, these results suggested that mechanical stress induced pro-inflammatory chemokines IL8 and CCL20 release by PDLSCs through eATP-P_2_X_7_ receptor interaction [55].

Mechanical stress is also involved in the maintenance of periodontium homeostasis by controlling the major pro-inflammatory mediator IL1*β* processing and release by PDLCs. Continuous compressive loading upregulated IL1*β* expression through the release of ATP in PDLCs. IL1*β* expression was markedly inhibited by a P_2_X_7_ receptor inhibitor or siRNA targeting the P_2_X_7_ receptor. As the P_2_X_7_ receptor is an ion channel receptor mostly permeable to calcium, intracellular calcium inhibitors markedly inhibited eATP-induced IL1*β* expression. According to this result, eATP-P_2_X_7_ receptor signalling and intracellular calcium signalling mechanisms are importantly imparted in mechanical stress-induced PDLCs inflammation through induction of pro-inflammatory cytokine, IL1 *β* production [12]. In next to the latter study, the role of pannexin1 (Panx1) in ATP-induced IL1 *β* expression in PDLCs was examined. The release of ATP is decreased by using a Panx1 inhibitor. Blocking Panx 1 also inhibited the release of IL1*β* which was induced by mechanical stress or ATP. Vesicular trafficking inhibitors reduced the release of IL1*β* by stimulated cells. Therefore, Panx-1 is contributed to the release of ATP and also to the release of IL1*β* induced by mechanical stress or ATP treatment [56].

### Effects of eATP and immunomodulatory function of PDLCs

The immunomodulatory function of PDLCs is very important in host immune responses by suppressing inflammation, initiating the repairing process, and getting efficient regeneration. eATP stimulates the immunomodulatory function of PDLCs by promoting immunomodulatory molecules IDO and IFN*γ* release. Inhibition of P_2_X_7_ receptor by using chemical P_2_X_7_ antagonists; BBG and KN62, siRNA targeting P_2_X_7_ receptor, calcium chelator (EGTA), and PKC inhibitor significantly reduced eATP-induced IDO and IFN*γ* expression. Specific P_2_X_7_ receptor agonists (BzATP) dramatically induced eATP induced these two molecules’ expression. Hence P_2_X_7_receptor activation and intracellular calcium signalling are related to an immunomodulatory property of PDLCs. The eATP takes part in the immunosuppressive action of PDLCs [57].

### Effects of eATP on osteogenic differentiation of PDLCs

One of the major functions of PDLCs is the differentiation function. eATP has also a key role in the maintenance of osteogenic differentiation of PDLCs. ATP-P_2_X_7_ receptor interaction decreases osteogenesis on inflammatory mediated PDLSCs through the PI_3_k-Akt-mTOR signalling pathway [58]. ATP enhances the osteogenic potential of PDLSCs by enhancing osteogenic genes Runx2 and OCN expression after 1 week of ATP treatment in an osteogenic medium. ATP treatment also demonstrated a highly expressed P_2_X_7_ receptor in PDLSCs. Moreover, ATP activates the P_2_X_7_ receptor, enhancing the PDLSCs osteogenesis [59]. PDLCs can differentiate into osteoblastic cells under cyclic tensile force. Continuous cyclic tensile force applied for 6 h stimulated osteogenic protein BMP9 synthesis and induced mineralization of PDLCs within 14 days of mineralization. Loss of function and overexpression experiments using suramin (a broad-spectrum P_2_Y antagonist), specific P_2_Y_1_ antagonist (MRS2179), or specific P_2_Y_1_ receptor agonist revealed the involvement of P_2_Y_1_ receptor in the induction of BMP9 synthesis. Experiments using U‐73122 (a phospholipase C [PLC] inhibitor), and thapsigargin (enhancer of intracytosolic calcium) also suggested the synthesis of BMP9 is related to an increased level of intracellular Ca^2+^ through the PLC pathway. These results indicated that eATP-P_2_Y_1_ signalling participated in CTF-induced BMP9 synthesis and in vitro mineralization [60]. Compressive including intermittent compressive force (ICF) and continuous compressive force (CCF) significantly increased extracellular ATP levels and ICF involved in the upregulation of osteogenic gene osterix expression through transforming growth factor *β* pathway. However, exogenous ATP treatment did not show an effect on the osteogenic differentiation of PDLCs [10].

### Effects of eATP on the bone-destructive function of PDLCs

Mechanical stress induced the release of ATP by PDLCS and also promotes osteopontin (OPN) expression in PDLCs via the Rho kinase pathway. Osteopontin is the protein involved in bone destruction. Mechanical stress-induced ATP upregulates OPN which is mediated by the P_2_Y_1_-Rho kinase signalling pathway. Therefore, stress-induced ATP plays part in alveolar bone destruction [61]. In another study, mechanical stress-induced ATP increased bone-destructive protein RANKL expression. Upregulation of RANKL expression was mediated by the same P_2_Y_1_ receptor activation but through a different pathway. Indomethacin (an inhibitor of COX), H89 (cAMP-dependent protein kinase inhibitor) and pyrrolidine dithiocarbamate (NF*κ*B inhibitor) inhibited RANKL expression, PGE2 production and NF*κ*B translocation. Thus, eATP participates in the maintenance of bone homeostasis mediated by the P_2_Y_1_-NF*κ*B-COX-RANKL axis in the periodontal tissue [62]. Therefore, eATP is related to bone homeostasis function by inducing different bone-destructive protein expressions through different signalling pathways.

### Effects of eATP on PDL repair

PDLCs are mechanosensitive cells, receiving mechanical stress from dental occlusion or orthodontic tooth movement. Mechanical stress like centrifuge-mediated gravity loading increased ATP in the extracellular environment and extracellular signal-regulated kinases (ERK) phosphorylation in PDLCs. ERK phosphorylation imparts in the remodelling of periodontal tissues. Gravity loading induced ATP release and ERK phosphorylation in PDLCs which in turn would enhance the growth and survival of PDLCs. Stress-induced-ATP is involved in the stimulation of periodontal tissue remodelling via the P_2_Y receptor especially P_2_Y_4_ and P_2_Y_6_ during orthodontic tooth movement [63].

### Effects of eATP on other functions of PDLCs

Continuous compressive stress causes the induction of ATP release by PDLCs. The mechanism of ATP release is dependent on the opening of hemichannel protein especially connexin 43. Also, this mechanism is regulated by the intracellular Ca^2+^ signalling pathway. Nevertheless, hemichannel gap junction proteins play important role in the function and behaviour of the PDLCs [11].

PDLCs respond to orthodontic tooth movement-related nociceptive pain by releasing ATP. Vesicular nucleotide transporter (VNUT) takes part in the uptake of ATP into secretory vesicles and this ATP binds to P_2_X_3_ receptor on trigerminal nerve resulting in tooth movement-induced pain. VNUT inhibitors (clodronic acid) suppressed the release of ATP induced by mechanical stimulation. Systemic administration of clodronic acid inhibited face-grooming behaviour (an indicator of nociception) followed by 1 day of experimental tooth movement. Moreover, ATP could regulate nociceptive pain control related to orthodontic tooth movement [64].

## Discussion And conclusion

Many previous studies proved that eATP has a variety of effects on the functions of different cell types. ATP acts as an intracellular source of energy as well as involved in various intracellular signalling events. Different cell types including PDLCs could release ATP into the extracellular environment in response to mechanical stimuli and inflammatory conditions, and the released ATP has a possibility to take part in different PDLCs’ functions. The released ATP by PDLCs acts as a danger signal that stimulates inflammatory reactions, interestingly it also involves in the immunosuppressive action of PDLCs suggesting its biphasic effects on the bone remodeling of PDLCs. Nevertheless, PDLCs are one of the key players in the homeostasis of the periodontium and PDLCs have various kinds of functions. The role of eATP on PDLCs’ functions and the detailed mechanism is lesser compared to other cell types, so many further studies are required to assess the effect of eATP on different PDLCs’ functions such as angiogenesis, differentiation, and their detailed mechanism that help to get future successful periodontal regeneration therapy.

The periodontal regeneration is a complicated process, and the final goal of periodontal regeneration is the removal of destructive tissue as well as the replacement of new functional structure. To fulfill this goal, PDLCs therapy is a priority for the regeneration but there are a lot of limitations; for example, inflammatory resident tissues that release inflammatory cytokines and change the regenerative ability of host tissue leading to poor prognosis of the cell treatment and failure. Therefore, adjunct strategies such as different growth factors, natural biomaterials are needed to induce cell homing, promote resident cell proliferation and differentiation, induce immunomodulation of host system, regulate cell signalling to get endogenous periodontal regeneration [65]. Nowadays, many studies found that different growth factors, signalling molecules, drugs used as adjuncts to conventional periodontal therapy. For example, local delivery of recombinant PDGF-BB using *β*TCP carrier promote periodontal wound healing by inducing the expression of ICTP, VEGF, PDGF [66]. Local application of recombinant FGF to infrabony defect improve alveolar bone growth [67]. Many growth factors and small molecules released by cells become target to improve regeneration process. According to the previous studies’ results, eATP may be a promising therapeutic tool for future periodontal regenerative therapy. The inductive and inhibition effect of eATP via different purinergic P_2_ receptor signaling may be applied in creating therapeutic material used as adjuncts for conventional periodontal therapy.

**Fig. 2:**
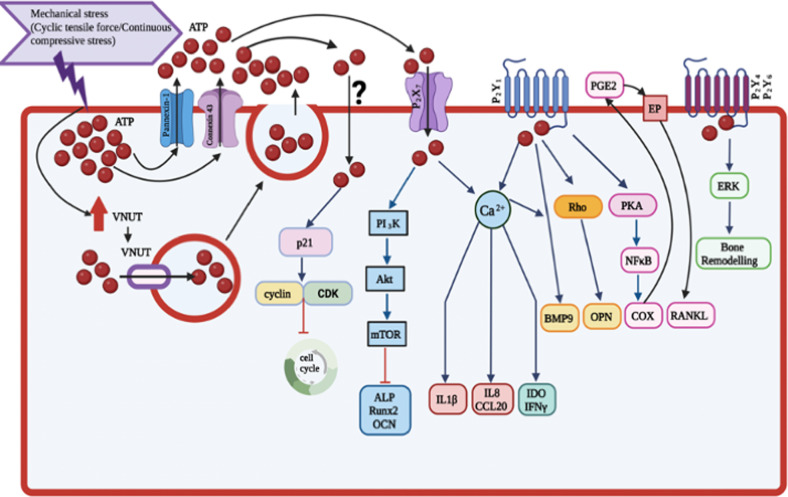
Effects of adenosine triphosphate on the functions of periodontal ligament cells. This figure summarized the effects of eATP-different P_2_ receptor signalling pathway in mechanically stimulated PDLCs (Created with BioRender.com).

Taken together, eATP plays important role in the control of pro-inflammatory cytokine and chemokine release, inhibition of proliferation, stimulating immunosuppressive action as well as inhibiting or stimulating osteogenic differentiation of PDLCs through various purinergic P_2_ receptors and signalling pathways (Fig. 2). These findings improve the knowledge about the released nucleotide ATP support PDLCs to regulate periodontal tissue homeostasis and regeneration process. Understanding the role of eATP on PDLCs functions beneficially applied for the development of new adjunct strategies for the periodontal healing process. With the addition of new advancing technologies, eATP may be utilized as a therapeutic molecule to improve future periodontal regeneration therapy as an adjunct molecule after periodontal surgery to improve the healing process of periodontal defect or used after scaling to control the progress of the periodontal disease. However, further studies are needed to extend the insight of eATP on the periodontal regeneration process.

## Appendix

Appendix 1 List of abbreviations

**Table:**

eATP	Extracellular adenosine triphosphate
ATP	Adenosine triphosphate
PDLCs	Periodontal ligament cells
VEGF	Vascular Endothelial Growth Factor
FGF2	Fibroblast growth factor 2
IL	Interleukin
PKC	Protein kinase C
PI_3_K	Phosphatidylinositol 3-kinase
Akt	Ak mouse strain transforming serine-threonine protein kinase
MAPK	Mitogen activated protein kinase
MG-5	Microglial cell line 5
IDO	Indoleamine-pyrrole 2,3-dioxygenase
BMSCs	Bone marrow mesenchymal stem cells
PRISMA	Preferred Reporting Items for Systematic Reviews and Meta Analyses
PDL	Periodontal ligament
PDLSCs	Periodontal ligament stem cells
rhBMP-2	Recombinant bone morphogenetic protein-2
PDGF	Platelet-derived growth factor
TGF-β1	Transforming growth factor β1
LPS	Lipopolysaccharides
IFNγ	Interferon-gamma
HLA-G	Human leukocyte antigen G
Poly I:C	polyinosinic-polycytidylic acid
TLR3	Toll like receptor 3
iATP	Intracellular adenosine triphosphate
TNFα	Tumor necrosis factor alpha
Mg	Magnesium
DAMP	Danger-Associated- Molecular-Pattern Molecule
P_2_	Purinergic 2 receptor
P_2_YRs	P_2_Y family of purinergic receptors
P_2_YRs	P_2_X family of purinergic receptors
ADP	Adenosine diphosphate
AMP	Adenosine monophosphate
UDP	Uridine diphosphate
UTP	Uridine triphosphate
NAD	Nicotinamide-adenine dinucleotide
PLC	Phospholipase C
IP_3_	Inositol triphosphate
AC	Adenylyl cyclase
cAMP	Cyclic adenosine monophosphate
K^+^	Potassium
Ca^2+^	Calcium
PKA	Protein kinase A
NALP3	NACHT, LRR and PYD domains-containing protein 3
*P. gingivalis*	*Porphyromonas gingivalis*
ERK	Extracellular signal-regulated kinases
p38	38-kDa protein
JNK	c-Jun N-terminal kinase
MCF-7	Breast cancer cell line acronym for Michigan Cancer Foundation-7
MMPs	Matrix metalloproteinases
CD	Cluster of differentiation
CD39	Ectonucleoside triphosphate diphosphohydrolase-1
CD^4+^ T cells	Helper T cells
Treg	Regulatory T cells
ATPγS	Slowly hydrolysable ATP
BzATP	2’(3’)-*O*-(4-Benzoylbenzoyl) adenosine-5’-triphosphate tri(triethylammonium) salt
CCL20	Chemokine (C-C motif) ligand 20
oATP	Oxidized adenosine triphosphate
A-74003	Artificial P_2_X_7_ receptor antagonist
siRNA	Small interfering RNA
Panx1	Pannaxin-1
BBG	Brilliant Blue G
KN62	4-[(2 S)-2-(N-Methylisoquinoline-5-sulfonamido)-3-oxo-3-(4-phenylpiperazin-1-yl) propyl] phenyl isoquinoline-5-sulfonate
EGTA	Ethylene glycol-bis(2-aminoethylether)-*N,N,N*′*,N*′-tetraacetic acid
mTOR	Mammalian target of rapamycin
BMP9	Bone morphogenetic protein 9
MRS2179	Competitive antagonist at P_2_Y_1_ receptors
U-73122	Phospholipase C and 5-lipooxygenase inhibitor
CTF	Cyclic tensile force
ICF	Intermittent compressive force
CCF	Continuous compressive force
OPN	Osteopontin
Rho	Ras homologous (Rho) protein
RANKL	Receptor activator of nuclear factor-kappa B ligand
COX	Cyclo-oxygenase
H-89	Potent and selective inhibitor of cyclic AMP-dependent protein kinase
NFκB	Nuclear factor kappa B
PGE2	Prostaglandin E2
VNUT	Vesicular nucleotide transporter
Runx2	Runt related transcription factor 2
OCN	Osteocalcin
*β*TCP	Beta tricalcium phosphate
ICTP	pyridinoline cross- linked carboxyterminal telopeptide of Type I collagen
